# Complexity Entropy Analysis of Grid Chaotic System: Image Encryption and DSP Implementation

**DOI:** 10.3390/e28060698

**Published:** 2026-06-16

**Authors:** Gang Hu, Baolin Kang, Xiaolin Ye

**Affiliations:** 1 School of Mathematics, Anshan Normal University, Anshan 114005, China; hugang@hrbeu.edu.cn; 2 School of Electronic and Information Engineering, University of Science and Technology Liaoning, Anshan 114051, China; yexl0207@163.com

**Keywords:** chaos, complexity, ADM, Lyapunov exponent

## Abstract

In this research, based on Adomian decomposition method (ADM), we construct true fractional-order differential equations. Due to the boosting function brought by the sine function, the system can output infinite coexistence attractors on *y*–*z* planes. In particular, this grid effect becomes increasingly obvious as the fractional order increases. Based on this boosting grid idea, in combination with the fractal dynamics, we construct some fractal patterns, e.g., Koch snows. These fractal diagrams all present grid fractal shapes. And then, we design a grid image encryption algorithm. This algorithm is proven to have higher security. The combination of chaos and fractals explores a new research direction. It provides new ideas for research in related fields.

## 1. Introduction

Multistable chaos has attracted much attention in recent research [1,2,3]. Due to directional and non-directional movement of fixed and eternal points, the power system can present multiple stabilities. In terms of a chaotic dynamical system, it usually can generate coexisting attractors or an infinite number of coexisting attractors [4,5,6], in particular by introducing trigonometric functions towards system models. Some primitive systems have the ability to coin infinitely many attractors on two planes. This special attractor is often referred to as the grid attractor [7,8]. Compared with ordinary single-stable systems, multi-stable systems exhibit more diverse dynamic behaviors. This work is worthy of studying.

The fractional system is an extension of integer-order calculus. Its core lies in extending the order of differential and integral operations from the integer field to the real number field and even the complex number field [9,10]. The uniqueness of a fractional-order system stems from its non-locality and memory characteristics. The fractional derivative depends on the historical information of the function throughout the entire time interval rather than just the current state, which makes the dynamical character closely related to the historical input [11]. The attenuation characteristics of its kernel functions directly affect the system response. For instance, in the phenomenon of abnormal diffusion, the root mean square of the particle displacement increases in a non-integer power law over time [12]. Fractional-order systems also exhibit complex nonlinear behaviors and dynamic diversity. Even simple systems may generate hidden attractors, multi-stability, and other phenomena [13,14,15]. This characteristic has important applications in fields such as chaos control [16] and secure communication [17]. Ref. [18] gives a system model of hyperchaotic memristive circuit and finds the phenomenon of coexisting evolution with different order *q*. Ref. [19] proposes an extremely simple fractional-order model: due to the boosting functions, the new equation model produces fractional-order multistable attractors. In our research, the fractional solution method we apply is ADM. Compared with several traditional algorithms, it shows higher numerical stability for the fractional-order model.

Differential systems form attractor patterns due to the superposition of multi-period evolution [20,21]. Each superposition of the chaotic sequence has self-similarity. Fractal dynamics has many similar characteristics to chaotic dynamics, especially self-similarity [22]. Essentially, it is because both are the results obtained through multiple iterations of differential or difference equations. That is to say, the iteration of the system produces this similarity [23]. Fractology is a branch of mathematics [24]. The term “fractal” was coined by Mandelbrot in 1975, which originated from the Latin word “fractus”, meaning “broken” or “fractured” [25]. Fractals are regarded as infinitely complex. If one keeps magnifying a fractal image, the same structure can always be seen. But creating a fractal is a simple process that only requires repeating the same process over and over again [7]. Recently, some new ideas have been proposed in the research of chaos and fractals. Ref. [26] gives a novel system equation of grid attractors and designs some graftals on basis of the Julia set. Ref. [27] proposes a different fractal process based on the Julia set, on the basis of some seed system model such as double-scroll or multi-scroll chaotic attractors, and gives some new graftals. Ref. [28] gives some fractal pattern based on a differential equation and designs some control strategy. Based on the extremely numerous similarities in dynamics between chaos and fractals, we propose a dynamic study based on the grid dynamics to combine fractional-order chaos and fractals.

This study focuses on the fractional-order grid, dynamical and fractal. It is summarized as follows. In Section 2, a fractional-order grid dynamical model is given. In Section 3, the grid fractal, e.g., Koch snows are given. In Section 4, a grid image encryption algorithm is designed.

## 2. Numerical Analysis of the Seed System

### 2.1. Adomian Decomposition Method

ADM is a mathematical tool used for solving the nonlinear differential equations and integral equations. The state variable is set as x(t). By using the Caputo operator, for differential equations, we have Equation (1).

(1) $$\left\{\begin{array}{c} {}*D_{t0}^{q}\left(t\right)=Lx\left(t\right)+Nx\left(t\right)+g\left(t\right)\\ x_{k}\left(t_{0}^{+}\right)=b_{k},k=0,1,\ldots ,m-1,\; \end{array}\right.$$

Here, Lx(t) and Nx(t) display the linear–nonlinear terms, and g(t) and $b_{k}$ are defined as constants. We can get Equation (2).

(2) $$x=J_{t0}^{q}Lx+J_{t0}^{q}Nx+J_{t0}^{g}+\sum_{k=0}^{m-1}b_{k}\frac{{\left(t-t_{0}\right)}^{k}}{k!},$$

in which $J_{t0}^{q}$ is defined as the Riemann–Liouville fractional operator and the interval range of time *t* is in $\left[t_{0},t_{1}\right]$. Ulteriorly, we have Equation (3).

(3) $$\left\{\begin{array}{c} J_{t0}^{q}{\left(t-t_{0}\right)}^{r}=\frac{\Gamma (r+1)}{\Gamma (r+1+q)}\\ J_{t0}^{q}C=\frac{C{\left(t-t_{0}\right)}^{q}}{\Gamma (q+1)}\\ J_{t0}^{q}J_{t0}^{r}x\left(t\right)=J_{t0}^{q+r}x\left(t\right),\; \end{array}\right.$$

in which the nonlinear terms of the previous formula can be resolved by Equation (4).

(4) $$\left\{\begin{array}{c} A_{j}^{i}=\frac{1}{i!}{\left[\frac{d^{i}}{d\lambda^{i}}N\left(v_{j}^{i}\left(\lambda \right)\right)\right]}_{\lambda =0}\\ v_{j}^{i}\left(\lambda \right)=\sum_{k=0}^{i}\lambda^{k}x_{j}^{k}\\ Nx=\sum_{i=0}^{\infty }A^{i}\left(x^{0},x^{1},\ldots x^{i}\right).\; \end{array}\right.$$

After the above formula calculations, a time series can be decomposed into the following parts as Equation (5):

(5) $$x=J_{t0}^{q}\sum_{i=0}^{\infty }x^{i}+J_{t0}^{q}\sum_{i=0}^{\infty }A^{i}+J_{t0}^{q}g+\sum_{k=0}^{m-1}b_{k}\frac{{\left(t-t_{0}\right)}^{k}}{k!}$$

For different time series $x^{i}$, according to ADM, they can be described as Equation (6).

(6) $$\left\{\begin{array}{c} x^{0}=J_{t0}^{q}g+\sum_{k=0}^{m-1}b_{k}\frac{{\left(t-t_{0}\right)}^{k}}{k!}\\ x^{1}=J_{t0}^{q}Lx^{0}+J_{t0}^{q}A^{0}\left(x^{0}\right)\\ x^{2}=J_{t0}^{q}Lx^{1}+J_{t0}^{q}A^{1}\left(x^{0},x^{1}\right)\\ \ldots \ldots \\ x^{i}=J_{t0}^{q}Lx^{i-1}+J_{t0}^{q}A^{i-1}\left(x^{0},x^{1},\ldots ,x^{i-1}\right)\; \end{array}\right.$$

Utilizing the above ADM, the approximate solution of the seed system can be given. Compared with other algorithms, ADM has its superiority for calculating the system’s solution.

### 2.2. Decompose the System by Using ADM

The seed system is a three-order continuous differential equation. Its characteristic is that the first two equations are independent terms. Such differential equations are the best choice for constructing grid attractors. By introducing two sine functions as excitation, its equation model is Equation (7).

(7) $$\left\{\begin{array}{c} \dot{x}=asin\left(y\right)\\ \dot{y}=bsin\left(z\right)\\ \dot{z}=-asin\left(y\right)-bcsin\left(z\right)-x+x^{2}.\; \end{array}\right.$$

According to ADM, its corresponding fractional mathematical model is Equation (8).

(8) $$\left\{\begin{array}{c} {}*\;D_{t0}^{q}x=asin\left(y\right)\\ {}*\;D_{t0}^{q}y=bsin\left(z\right)\\ {}*\;D_{t0}^{q}z=-asin\left(y\right)-bcsin\left(z\right)-x+x^{2},\; \end{array}\right.$$

Here, *q* shows its order, *a* and *b* are parameters. On the basis of ADM, the original integer-order system can be decomposed into Equation (9).

(9) $$\left\{\begin{array}{c} x_{n+1}=x_{n}+asin\left(y_{n}\right)\frac{h^{q}}{\Gamma (q+1)}+absin\left(sin\left(z_{n}\right)\right)\frac{h^{2q}}{\Gamma (2q+1)}+\ldots \\ y_{n+1}=y_{n}+bsin\left(z_{n}\right)\frac{h^{q}}{\Gamma (q+1)}+bsin\left(-asin\left(y_{n}\right)-bcsin\left(z_{n}\right)-x_{n}+x_{n}^{2}\right)\frac{h^{2q}}{\Gamma (2q+1)}+\ldots \\ z_{n+1}=z_{n}+\left(-asin\left(y_{n}\right)-bcsin\left(z_{n}\right)-\ldots \right)\frac{h^{q}}{\Gamma (q+1)}+\left(-absin\left(\ldots \right)+\ldots \right)\frac{h^{2q}}{\Gamma (2q+1)}+\ldots \; \end{array}\right.$$

where Γ() and *h* define the Gamma function. And then, we can get a fifth-order iterative equation system as follows in Equations (10)–(16).

(10) $$\left\{\begin{array}{c} C_{10}=x_{n}\\ C_{20}=y_{n}\\ C_{30}=z_{n}\; \end{array}\right.$$

(11) $$\left\{\begin{array}{c} C_{11}=asin\left(C_{20}\right)\\ C_{21}=bsin\left(C_{30}\right)\\ C_{31}=-asin\left(C_{20}\right)-bcsin\left(C_{30}\right)-C_{10}+C_{10}^{2}\; \end{array}\right.$$

(12) $$\left\{\begin{array}{c} C_{12}=asin\left(C_{21}\right)\\ C_{22}=bsin\left(C_{31}\right)\\ C_{32}=-asin\left(C_{21}\right)-bcsin\left(C_{31}\right)-C_{11}+2C_{10}C_{11}\; \end{array}\right.$$

(13) $$\left\{\begin{array}{c} C_{13}=asin\left(C_{22}\right)\\ C_{23}=bsin\left(C_{32}\right)\\ C_{33}=-asin\left(C_{22}\right)-bcsin\left(C_{32}\right)-C_{12}+2C_{10}C_{12}+C_{11}^{2}\frac{\Gamma (2q+1)}{\Gamma^{2}\left(q+1\right)}\; \end{array}\right.$$

(14) $$\left\{\begin{array}{c} C_{14}=asin\left(C_{23}\right)\\ C_{24}=bsin\left(C_{33}\right)\\ C_{34}=-asin\left(C_{23}\right)-bcsin\left(C_{33}\right)-C_{13}+2C_{10}C_{13}+2C_{11}C_{12}\frac{\Gamma (3q+1)}{\Gamma (q+1)\Gamma (2q+1)}\; \end{array}\right.$$

(15) $$\left\{\begin{array}{c} C_{15}=asin\left(C_{24}\right)\\ C_{25}=bsin\left(C_{34}\right)\\ C_{35}=-asin\left(C_{24}\right)-bcsin\left(C_{34}\right)-C_{14}+2C_{10}C_{14}+2C_{11}C_{13}\frac{\Gamma (4q+1)}{\Gamma (q+1)\Gamma (3q+1)}+\\ 2C_{12}C_{12}\frac{\Gamma (4q+1)}{\Gamma {\left(2q+1\right)}^{2}}\; \end{array}\right.$$

(16) $$\left[\begin{array}{c} x_{n+1}\\ y_{n+1}\\ z_{n+1} \end{array}\right]=\left[\begin{array}{c} C_{10}\;\;C_{11}\;\;C_{12}\;\;C_{13}\;\;C_{14}\;\;C_{15}\\ C_{20}\;\;C_{21}\;\;C_{22}\;\;C_{23}\;\;C_{24}\;\;C_{25}\\ C_{30}\;\;C_{31}\;\;C_{32}\;\;C_{33}\;\;C_{34}\;\;C_{35} \end{array}\right]={\left[\begin{array}{c} 1\;\;\frac{\Gamma (h^{q})}{\Gamma (q+1)}\;\;\frac{\Gamma (h^{2q})}{\Gamma (2q+1)}\;\;\frac{\Gamma (h^{3q})}{\Gamma (3q+1)}\;\;\frac{\Gamma (h^{4q})}{\Gamma (4q+1)}\;\;\frac{\Gamma (h^{5q})}{\Gamma (5q+1)} \end{array}\right]}^{T}$$

**Figure 1:**
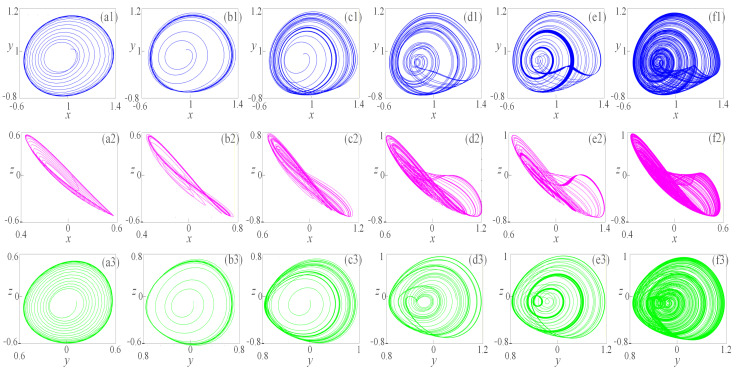
Chaotic attractors with different order *q* on different planes. (**a1**–**a3**) Chaotic attractors when *q* = 0.35. (**b1**–**b3**) Chaotic attractors when *q* = 0.45. (**c1**–**c3**) Chaotic attractors when *q* = 0.55. (**d1**–**d3**) Chaotic attractors when *q* = 0.75. (**e1**–**e3**) Chaotic attractors when *q* = 0.85. (**f1**–**f3**) Chaotic attractors when *q* = 0.95.

According to fifth-order ADM, the fractional-order system’s approximate solution can be found. We take control parameters *a* = 1, *b* = 1.35 and *c* = 0.37. The original state is [0.1, 0.1, 0.1], and sp the attractors with different order *q* can be given. As given by Figure 1, the chaotic attractors with different order *q* on different planes can be shown. From the phase plane trajectory given by Figure 1, with *q* increases, the chaos characteristic also gradually intensifies.

### 2.3. Grid Coexistence Attractors Based on the Seed System

**Figure 2:**
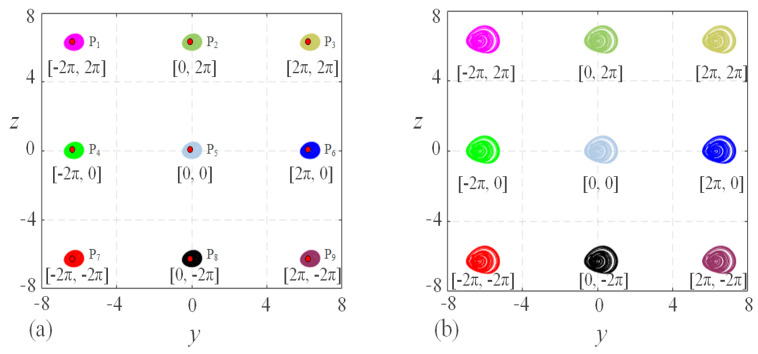
Fractional -order grid attractors. (**a**) Grid attractors when *q* = 3.5. (**b**) Grid attractors when *q* = 9.5.

Due to the unique structural features of the seed system, after introducing two sine functions, the newly constructed system can generate great many stable states. We slect control parameters *a* = 1, *b* = 1.35 and *c* = 0.37. The original state is [0.1, $y_{0}$, $z_{0}$], $y_{0}$ and $z_{0}$ are two variables. Figure 2a shows fractional-order grid coexistence attractors when the orders *q* = 3.5. $P_{1}$∼$P_{9}$ are the corresponding equilibrium points. As the equilibrium points keeps shifting, the position of the attractor presents a grid pattern. As given by Figure 2b when the order of fractional-order seed system *q* = 9.5, its corresponding grid coexistence attractors are shown. The increase in order leads to an enhancement of the chaotic attractors. This interesting phenomenon deserves further exploration of its dynamic characteristics.

### 2.4. Grid Dynamics Based on the Seed System

**Figure 3:**
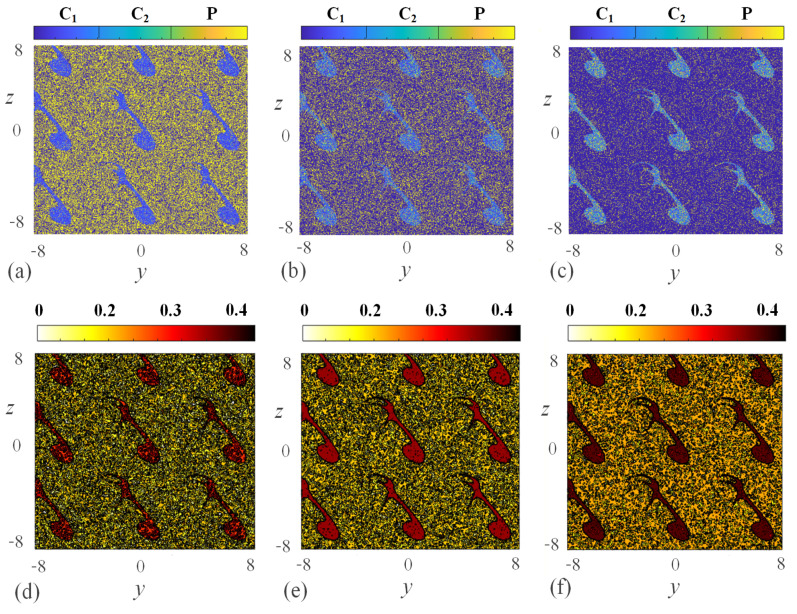
Fractional -order grid dynamics. (**a**) Grid attractor basin when *q* = 7.5. (**b**) Grid attractor basin when *q* = 8.5. (**c**) Grid attractor basin when *q* = 9.5. (**d**) Grid SE complexity when *q* = 7.5. (**e**) Grid SE complexity when *q* = 8.5. (**f**) Grid SE complexity when *q* = 9.5.

The attractor basin is a core concept in dynamical system theory, referring to the set of all initial points in the phase space that eventually converge to a specific attractor. Its classification is mainly based on the properties and numerical identifiability of attractors. Keeping the above parameters unchanged, Figure 3a–c give the fractional-order attractor basin of grid attractors when the order *q* = 7.5, 8.5 and 9.5, respectively. As order *q* increases, the chaos in the attraction basin also gradually intensifies.

Spectral entropy is an indicator for measuring the complexity of a signal spectral distribution. It gives the distribution characteristics by calculating the entropy value of the signal power spectral density function. The following is the calculation formula of spectral entropy and its explanation as Equation (17):

(17) $$H\left(f\right)=-\Sigma [P\left(f\right)\log_{2}P\left(f\right)\Delta f]$$

where P(f) represents the energy distribution of the signal at various frequencies. The higher the power spectral density, the greater the energy of the signal at that frequency. Δf is a discrete interval in the frequency domain, which determines the fineness of the spectral analysis. We sum up the entropy values of all frequency components and take a negative sign. The result obtained in this way is spectral entropy, which reflects the complexity of the signal’s spectral distribution. Spectral entropy is of great significance in signal processing and communication system design. Its specific applications include:

1. Signal spectrum analysis: By calculating the spectral entropy of the signal, the spectral distribution characteristics of the signal can be analyzed, which helps with frequency domain feature extraction and analysis.
2. Evaluation of spectral distribution uniformity: Spectral entropy can assess the uniformity of a signal’s distribution in the frequency domain, which is of great significance for spectral utilization and spectral allocation.
3. Modulation and demodulation of signals: In digital communication systems, by analyzing the spectral entropy of signals, appropriate modulation and demodulation methods can be selected to enhance the transmission rate and anti-interference capability of signals.

By using the SE algorithm based on the sequence of seed system, we can obtain its corresponding two-dimensional SE complexity simulation diagram. As given by Figure 3d–f, as  order *q* increases, the value of SE complexity also increases accordingly. From  the presented result given in Figure 3, it is similar to the result presented in Figure 2.

## 3. Grid Fractal Dynamical

### Grid Boosting Koch Snow Curve

The Koch snow curve is a classic fractal geometric figure. The construction method of the Koch snowflake curve is based on an equilateral triangle, and more complex shapes are generated by performing specific iterative operations on each side. We divide each edge into three equal parts. We draw an equilateral triangle outward from the middle one-third part, and then remove the base of this part. We repeat the above process for each new edge infinitely. The number of iterations is denoted by *n*. Set $x_{n}\to x_{n+1}$ represents the iterative process from this current graphic to the next one. The nth sequence $x_{n}$ = [$x_{1}$, $x_{2}$, …, $x_{k_{n}}$], where there are several points represented by $k_{n}$. For examole, when n=1, it represents a regular triangle. The complexity $x_{1}$ = [$x_{1}$, $x_{2}$, $x_{3}$, $x_{1}$] = [0, 1, 1/2(1+$\sqrt{3}i$), 0]. The reason for using the plural form is to facilitate mapping each section. We generate the grid Koch snow curve based on triangle bases according to the following process:

1. Divide each side of a regular triangle into three equal parts. The first and fifth points remain the same as before.
2. Add the second point at the 1/3 location original vector $x_{1}\to x_{2}$.
3. Add the third point, keep the length unchanged of 1/3 location original vector and turning it in the clockwise direction π/3.
4. Add the fourth point at the 1/3 location original vector $x_{1}\to x_{2}$.

**Figure 4:**
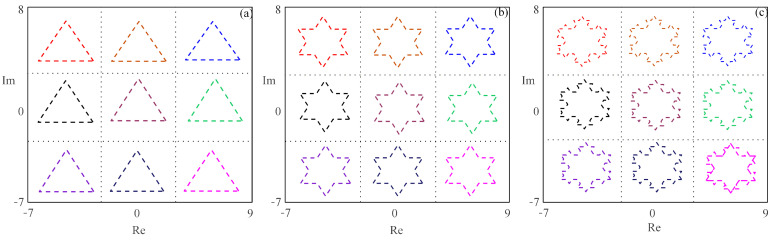

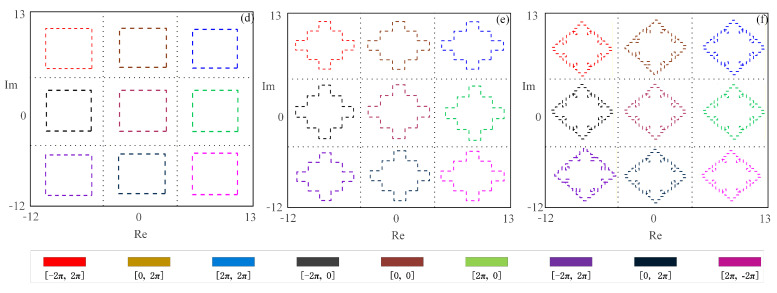
Grid Koch snow curve. (**a**) Koch snow curve based on triangle bases when the iterations = 0. (**b**) Koch snow curve based on triangle bases when iterations = 1. (**c**) Koch snow curve based on triangle bases when iterations = 2. (**d**) Koch snow curve based on square bases when iterations = 0. (**e**) Koch snow curve based on square bases when iterations = 1. (**f**) Koch snow curve based on square bases when iterations = 2.

The above algorithm can be solved in polar coordinates; the specific algorithm is shown in Algorithm 1. Homoplastically, we can obtain the grid Koch snow curve based on square bases. We select parameters *a* = 1, *b* = 1.35 and *c* = 0.37. The original state is [0.1, 0.1, 0.1], the order *q* = 0.95. We select the boosting sequences *y* and *z* of the fractional-order seed system as the input variable. Due to the boosting characteristics of sequences *y* and *z*, with the original state set as −2π∼2π and according to Algorithm 1, we can obtain the grid boosting Koch snow curve as given in Figure 4. In the figure, different colors represent Koch snow curves formed by different initial values. The structure of the grid boosting Koch snow curve enriches fractal dynamics.

**Algorithm 1:** Generate Koch snow curve

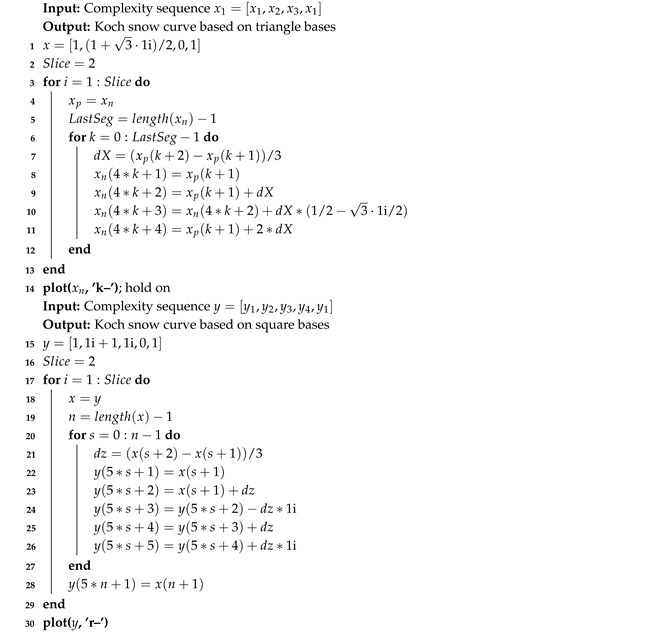



## 4. Encryption Process on of Fractal Basis

### Diffusion Encryption Scheme

**Figure 5:**
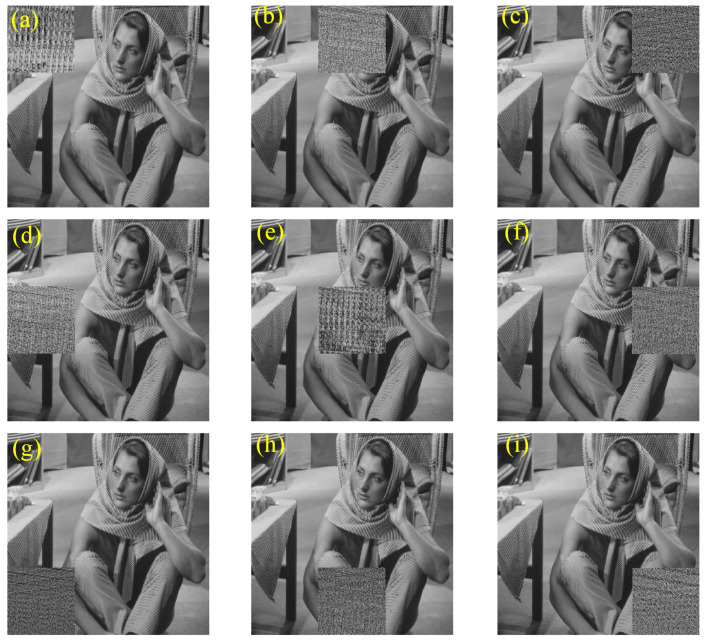
Grid Arnold Cat confusion encryption. (**a**) The Arnold confusion in the first area. (**b**) The Arnold confusion in the second area. (**c**) Arnold confusion in the third area. (**d**) Arnold confusion in the fourth area. (**e**) Arnold confusion in the fifth area. (**f**) Arnold confusion in sixth area. (**g**) The Arnold confusion in seventh area. (**h**) Arnold confusion in eighth area. (**i**) Arnold confusion in ninth area.

**Figure 6:**
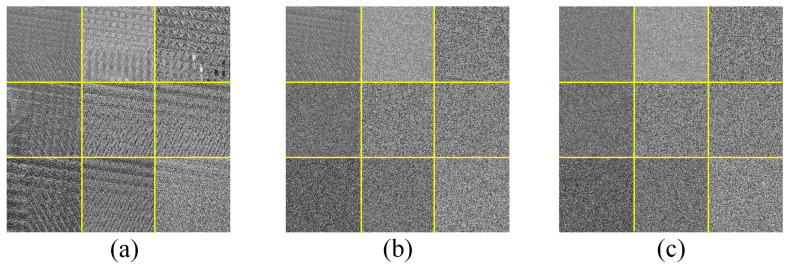
Grid Arnold Cat confusion effect. (**a**) One-time scrambling effect. (**b**) The scrambling effects of 1, 2, 3, …, 9 times, respectively. (**c**) Multi-times scrambling effect.

A chaotic equation is used as a pseudo-random number generator (PRNG) as part of the components in algorithm design. Particularly, we propose a new Arnold transformation as follows in Equation (18):

(18) $$\left[\begin{array}{c} x\\ y \end{array}\right]=\left[\begin{array}{cc} 1 & \;\;\;\;\;\;q\\ p & \;\;\;\;\;\;1+pq \end{array}\right]\left[\begin{array}{c} X-x_{0}\\ Y-y_{0} \end{array}\right]mod\left[\begin{array}{c} m\\ n \end{array}\right],$$

where $x_{0}$ and $y_{0}$ are the offset. After image testing, the image is given as in Figure 5 and Figure 6. On the basis of Equations (19)–(21), $u_{n}$, $v_{n}$ are the values of Algorithm 1. A new RGB three-channel diffusion schemes are as follows:

(19) $$\left\{\begin{array}{cc} RE_{1}\left(1\right) & =mod(R\left(1\right)+10^{5}\times k\times R\left(1\right)-10^{5}\times R\left(1\right)-floor\left(u_{1}\times 10^{15}\right),256)\\ RE_{1}\left(2\right) & =mod(R\left(2\right)+10^{5}\times k\times R\left(2\right)-10^{5}\times R\left(1\right)-floor\left(u_{2}\times 10^{15}\right),256)\\ RE_{1}\left(i\right) & =mod(R\left(i\right)+10^{5}\times k\times RE_{1}\left(i-1\right)-10^{5}\times RE_{1}\left(i-2\right)-floor\left(u_{i}\times 10^{15}\right),256)\\ GE_{1}\left(1\right) & =mod(G\left(1\right)+10^{5}\times k\times G\left(1\right)-10^{5}\times G\left(1\right)-floor\left(u_{1}\times 10^{15}\right),256)\\ GE_{1}\left(2\right) & =mod(G\left(2\right)+10^{5}\times k\times G\left(2\right)-10^{5}\times G\left(1\right)-floor\left(u_{2}\times 10^{15}\right),256)\\ GE_{1}\left(i\right) & =mod(G\left(i\right)+10^{5}\times k\times GE_{1}\left(i-1\right)-10^{5}\times GE_{1}\left(i-2\right)-floor\left(u_{i}\times 10^{15}\right),256)\\ BE_{1}\left(1\right) & =mod(B\left(1\right)+10^{5}\times k\times B\left(1\right)-10^{5}\times B\left(1\right)-floor\left(u_{1}\times 10^{15}\right),256)\\ BE_{1}\left(2\right) & =mod(B\left(2\right)+10^{5}\times k\times B\left(2\right)-10^{5}\times B\left(1\right)-floor\left(u_{2}\times 10^{15}\right),256)\\ BE_{1}\left(i\right) & =mod(B\left(i\right)+10^{5}\times k\times BE_{1}\left(i-1\right)-10^{5}\times BE_{1}\left(i-2\right)-floor\left(u_{i}\times 10^{15}\right),256) \end{array}\right.$$

where R(i)∼B(i) are the data of 3 channels, *k* = 30 is a variable, *u*, *v* are PRNs output by Algorithm 1. And we have the next diffusion formula:

(20) $$\left\{\begin{array}{c} RE_{2}\left(1\right)=mod(RE_{1}\left(1\right)-10^{6}\times k\times RE_{1}\left(1\right)-floor\left(RE_{1}{\left(1\right)}^{6}/255\right)\\ \times \left(1-RE_{1}\left(1\right)/255\right))+floor\left(v_{1}\times 10^{15}\right),256)\\ RE_{2}\left(2\right)=mod(RE_{1}\left(2\right)-10^{6}\times E_{0}\times RE_{1}\left(2\right)-floor\left(RE_{1}{\left(1\right)}^{2}/255\right)\\ \times \left(1-RE_{1}\left(1\right)/255\right))+floor\left(v_{2}\times 10^{15}\right),256)\\ RE_{2}\left(i\right)=mod(RE_{1}\left(i\right)-10^{6}\times k\times RE_{2}\left(i-1\right)-floor\left(RE_{2}{\left(i-2\right)}^{2}/255\right)\\ \times \left(1-RE_{2}\left(i-2\right)/255\right))+floor\left(v_{i}\times 10^{15}\right),256)\\ GE_{2}\left(1\right)=mod(GE_{1}\left(1\right)-10^{6}\times k\times GE_{1}\left(1\right)-floor\left(GE_{1}{\left(1\right)}^{6}/255\right)\\ \times \left(1-GE_{1}\left(1\right)/255\right))+floor\left(v_{1}\times 10^{15}\right),256)\\ GE_{2}\left(2\right)=mod(GE_{1}\left(2\right)-10^{6}\times E_{0}\times GE_{1}\left(2\right)-floor\left(GE_{1}{\left(1\right)}^{2}/255\right)\\ \times \left(1-GE_{1}\left(1\right)/255\right))+floor\left(v_{2}\times 10^{15}\right),256)\\ GE_{2}\left(i\right)=mod(GE_{1}\left(i\right)-10^{6}\times k\times GE_{2}\left(i-1\right)-floor\left(GE_{2}{\left(i-2\right)}^{2}/255\right)\\ \times \left(1-GE_{2}\left(i-2\right)/255\right))+floor\left(v_{i}\times 10^{15}\right),256)\\ BE_{2}\left(1\right)=mod(BE_{1}\left(1\right)-10^{6}\times k\times BE_{1}\left(1\right)-floor\left(BE_{1}{\left(1\right)}^{6}/255\right)\\ \times \left(1-BE_{1}\left(1\right)/255\right))+floor\left(v_{1}\times 10^{15}\right),256)\\ BE_{2}\left(2\right)=mod(BE_{1}\left(2\right)-10^{6}\times E_{0}\times BE_{1}\left(2\right)-floor\left(BE_{1}{\left(1\right)}^{2}/255\right)\\ \times \left(1-BE_{1}\left(1\right)/255\right))+floor\left(v_{2}\times 10^{15}\right),256)\\ BE_{2}\left(i\right)=mod(BE_{1}\left(i\right)-10^{6}\times k\times BE_{2}\left(i-1\right)-floor\left(BE_{2}{\left(i-2\right)}^{2}/255\right)\\ \times \left(1-BE_{2}\left(i-2\right)/255\right))+floor\left(v_{i}\times 10^{15}\right),256),\; \end{array}\right.$$

where $RE_{1}\left(i\right)$, $GE_{1}\left(i\right)$, and $BE_{1}\left(i\right)$ are pixel on the basis of 2-round diffusion. We let *k* = 40, $E_{0}$ = 45. And we have

(21) $$\left\{\begin{array}{cc} RE_{3}\left(1\right) & =RE_{1}\left(1\right)⊕RE_{2}\left(1\right)⊕mod\left(m_{1},256\right)\\ RE_{3}\left(2\right) & =RE_{1}\left(2\right)⊕RE_{2}\left(2\right)⊕mod\left(m_{2},256\right)\\ RE_{3}\left(i\right) & =RE_{1}\left(i\right)⊕RE_{2}\left(i\right)⊕mod\left(m_{i},256\right)\\ GE_{3}\left(1\right) & =GE_{1}\left(1\right)⊕GE_{2}\left(1\right)⊕mod\left(m_{1},256\right)\\ GE_{3}\left(2\right) & =GE_{1}\left(2\right)⊕GE_{2}\left(2\right)⊕mod\left(m_{2},256\right)\\ GE_{3}\left(i\right) & =GE_{1}\left(i\right)⊕GE_{2}\left(i\right)⊕mod\left(m_{i},256\right)\\ BE_{3}\left(1\right) & =BE_{1}\left(1\right)⊕BE_{2}\left(1\right)⊕mod\left(m_{1},256\right)\\ BE_{3}\left(2\right) & =BE_{1}\left(2\right)⊕BE_{2}\left(2\right)⊕mod\left(m_{2},256\right)\\ BE_{3}\left(i\right) & =BE_{1}\left(i\right)⊕BE_{2}\left(i\right)⊕mod\left(m_{i},256\right) \end{array}\right.$$

**Figure 7:**
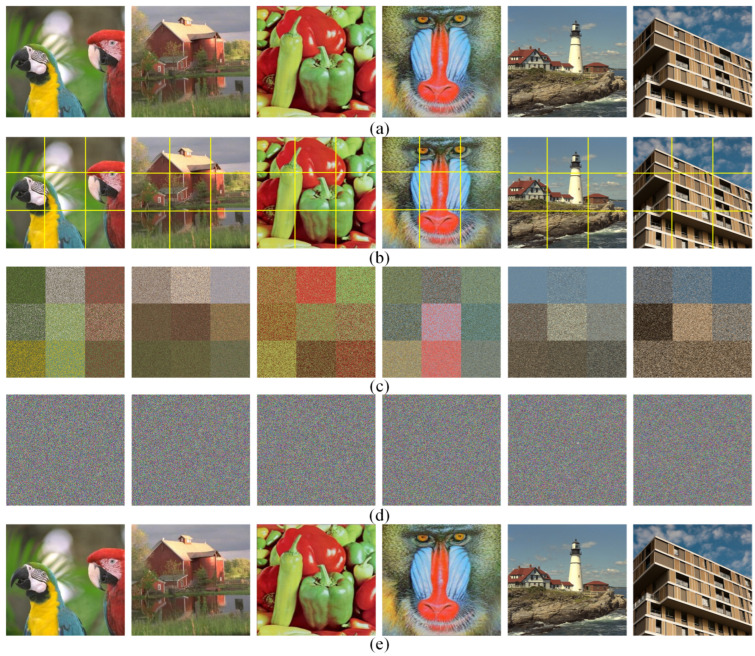
Encryption test. (**a**) Original P. (**b**) P after grid scheme. (**c**) P after grid Arnold scheme. (**d**) P after diffusion. (**e**) Decryption P.

**Figure 8:**
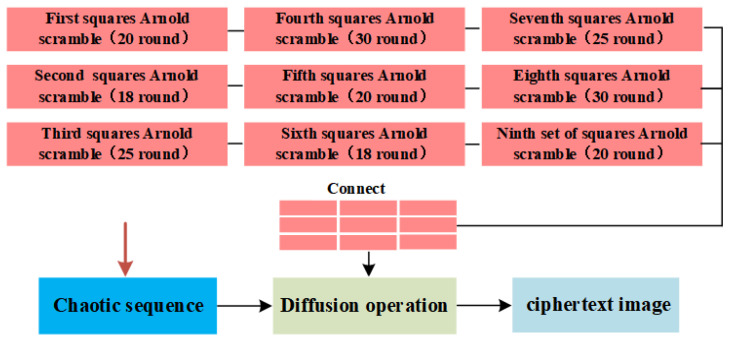
Encryption flowchart.

According to the encryption process, we use images from the Kodak dataset and have the ciphertext as shown by Figure 7. That original test image can be encrypted using the grid encryption method. The ciphertext image is given. And all the process is given in Figure 8.

## 5. Security Analysis

### 5.1. Testing by Chi-Square

$\chi^{2}$ is often applied as a testing tool in the quality analysis of cryptographic security. Its eq can be expressed as Equation (22):

(22) $$\begin{array}{c} \chi^{2}=\sum_{i=0}^{255}\frac{{\left(z_{i}-N/256\right)}^{2}}{N/256} \end{array}$$

Here, zi gives the occurrence frequency within a pixel. The ideal value is thought as 293.248. Table 1 gives the testing data by the design encryption scheme. The test results meet the high safety standards.

### 5.2. Histogram and Correlation

Image relevance is an important evaluation criterion in the field of computer vision. Its eq is Equation (23):

(23) $$\begin{array}{c} r_{xy}=\frac{\sum_{i=1}^{N}\left(x_{i}-\frac{1}{N}\sum_{i=1}^{N}x_{i}\right)\left(y_{i}-\frac{1}{N}\sum_{i=1}^{N}y_{i}\right)}{\sqrt{\sum_{i=1}^{N}{\left(x_{i}-\frac{1}{N}\sum_{i=1}^{N}x_{i}\right)}^{2}}\sqrt{\sum_{i=1}^{N}{\left(y_{i}-\frac{1}{N}\sum_{i=1}^{N}y_{i}\right)}^{2}}}. \end{array}$$

**Figure 9:**
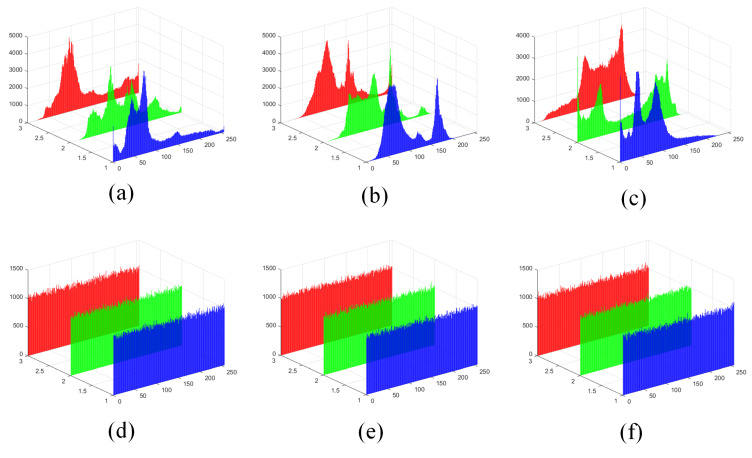

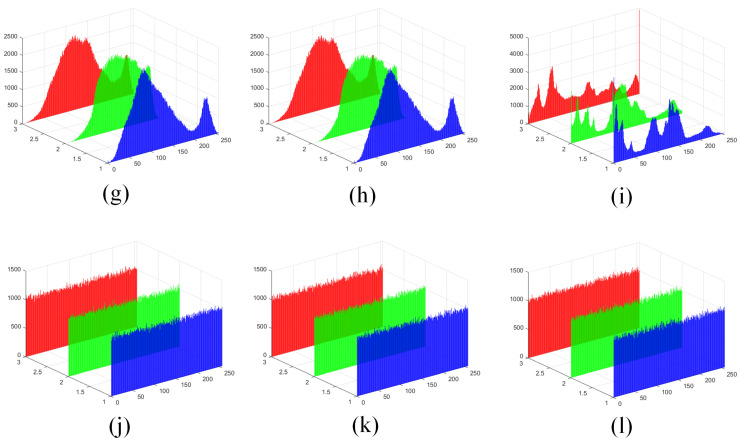
Histogram. (**a**) Histogram of PC1. (**b**) Histogram of PC2. (**c**) Histogram of PC3 (**d**) Histogram of CC1. (**e**) Histogram of CC2. (**f**) Histogram of CC3. (**g**) Histogram of PC4. (**h**) Histogram of PC5. (**i**) Histogram of PC6. (**j**) Histogram of CC4. (**k**) Histogram of CC5. (**l**) Histogram of CC6.

**Figure 10:**
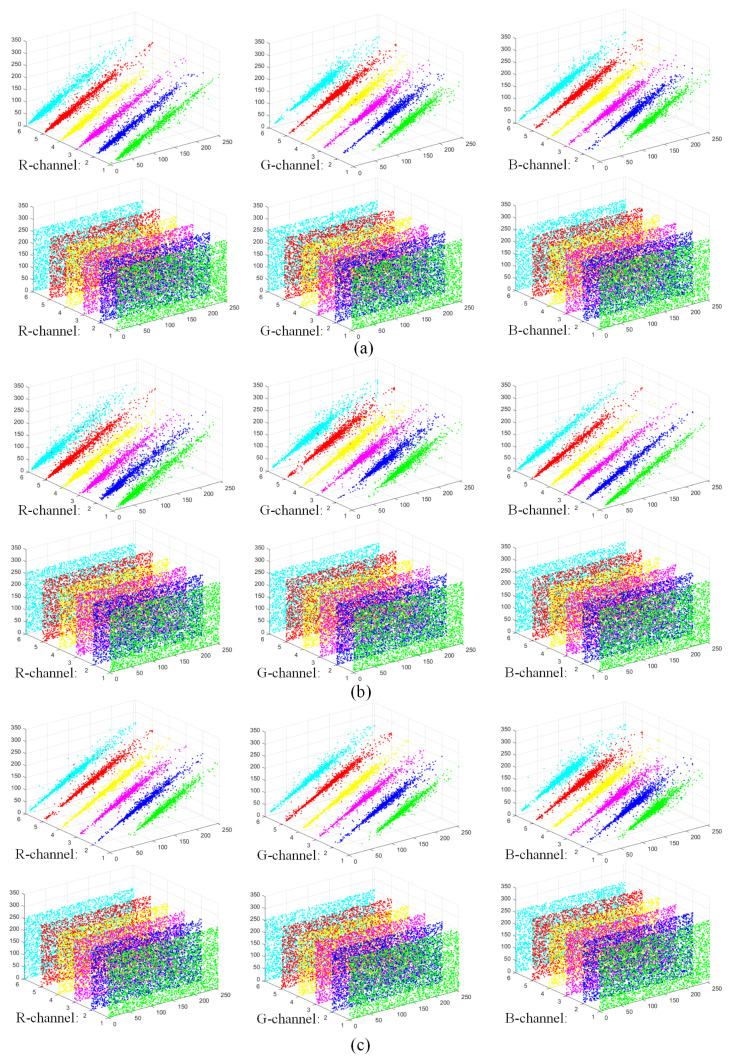
Correlation diagrams. (**a**) The correlation between plaintext and ciphertext on the horizontal direction. (**b**) The correlation between plaintext and ciphertext on the vertical direction. (**c**) The correlation between plaintext and ciphertext on the diagonal direction.

Based on the aforementioned algorithm design, tests were conducted on image correlation. Figure 9 presents test picture histogram. To provide more comparative data, the corresponding correlation was also tested in Figure 10. Table 2 presents the correlation coefficients.

### 5.3. Information Entropy and Robustness

The concept of entropy originated from thermodynamics. In thermodynamics, entropy is defined as the logarithm of the number of possible states of a system. In information theory, the output of the information source is a random quantity, and thus its uncertainty can be measured by a probability distribution. Its eq can be expressed as Equation (24):

(24) $$H\left(k\right)=\sum_{i=1}^{N}p\left(k_{i}\right)\log_{2}\frac{1}{p(k_{i})},$$

where relevant data are presented in Table 3 and Table 4. These data indicate that the information entropy of the encryption scheme is higher.

### 5.4. Anti-Differential Test

**Figure 11:**
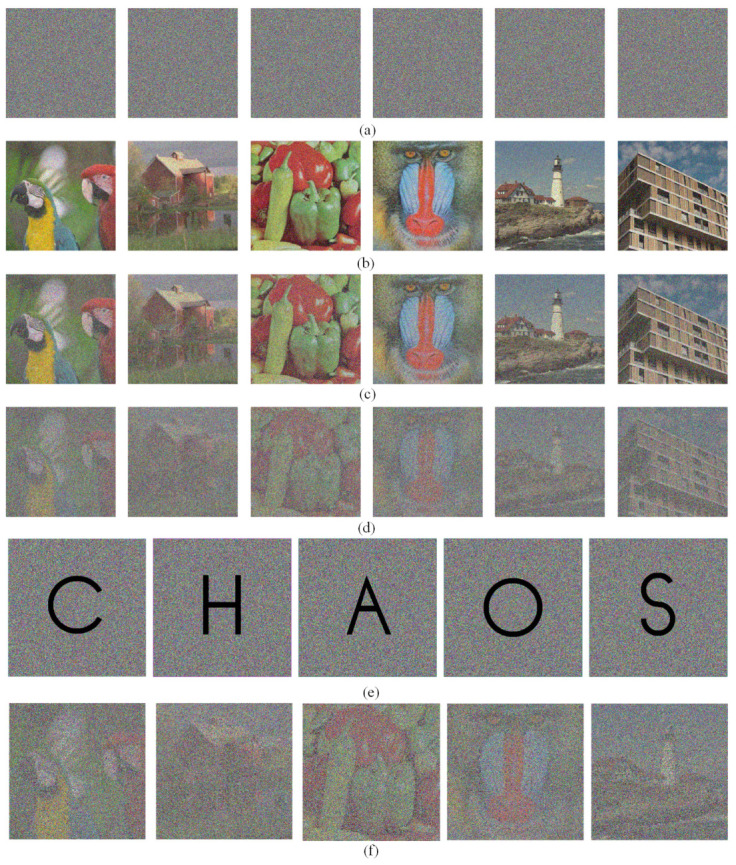
Robustness tests. (**a**) The salt and pepper noise attack is applied to the ciphertext. (**b**) The decrypted image with a density of 0.01% noise attack. (**c**) The decrypted image with a density of 0.02% noise attack. (**d**) The decrypted image with a density of 0.05% noise attack. (**e**) The shear attack is applied to the ciphertext. (**f**) The decrypted image with shear attack.

In this section, we use NPCR and UACI to test the security of the encryption scheme, shown in Equation (25):

(25) $$\left\{\begin{array}{c} NPCR=\frac{\sum_{i,j}D\left(i,j\right)}{L}\times 100\%\\ UACI=\frac{1}{L}\sum_{i,j}\frac{|C\left(i,j\right)-C_{1}\left(i,j\right)|}{256}\times 100\% \end{array}\right.,$$

where C1, C2 are the size of two images. Table 5 and Table 6 present the NPCR and UACI tests for the images, including black-only images, white-only images, and others. Table 7 gives the comparison data. The designed encryption scheme offers better security. Peculiarly, Figure 11 shows the test results of the noise attack and robustness analysis.

## 6. Hardware Implementation

**Figure 12:**
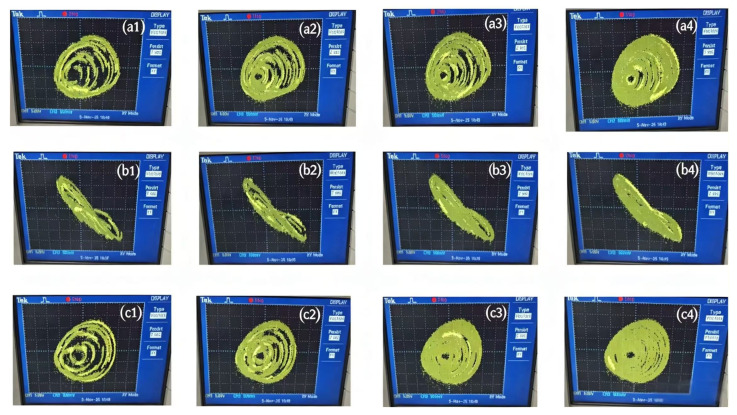
Attractors generated under the laboratory environment. (**a1**–**a4**) Attractor on *x*–*y* plane obtained by oscilloscope. (**b1**–**b4**) Attractor on *x*-*z* plane obtained by oscilloscope. (**c1**–**c4**) Attractor on *y*–*z* plane obtained by oscilloscope.

The system is physically realized by DSP, the main chip type is TMS320F28335. By applying ODE45, the numerical solution of the seed system can be obtained, and the attractors on each quadrant can be given. Figure 12 shows the realization of chaotic attractor on different planes by DSP.

## 7. Conclusions

In this research, we construct a 3D true fractional-order system on the basis of ADM. After the fifth-order decomposition, due to extreme multi-stability, the fractional-order system can produce an infinite number of coexistence attractors. By applying the sequences produced by the seed fractional-order chaotic system as the input, some grid fractal patterns are constructed, e.g., Koch snows and the Julia set. There are still some issues worth discussing in future research: Can the grid effect be produced by using other fractional construction methods? Besides the Julia set, there are others that can be used to generate grid fractal patterns. Next, we will continue to explore the dynamical intersection of chaos and fractals.

## Figures and Tables

**Table 1:** $\chi^{2}$ test.

Images	Original			Cipher		
	R	G	B	R	G	B
Color.1	233,289.501	163,124.562	305,041.553	296.851	284.556	282.994
Color.2	295,990.226	258,192.918	392,105.531	221.492	295.689	262.836
Color.3	212,151.843	118,044.902	331,064.697	270.945	231.978	282.744
Color.4	118,359.019	190,286.043	127,072.816	251.447	244.527	249.920
Color.5	337,860.513	357,685.906	788,927.957	247.873	225.978	239.771
Color.6	785,308.398	140,842.226	228,236.891	210.469	269.418	293.103

**Table 2:** Correlation coefficients.

Image	Image	Original			Cipher		
Name	Dire.	R	G	B	R	G	B
C1	H	0.9952	0.9941	0.9946	−0.0016	0.0007	−0.0026
512 × 512	V	0.9953	0.9939	0.9947	0.0006	−0.0005	0.0035
	D	0.9924	0.9904	0.9914	0.0035	−0.0028	0.0014
C2	H	0.9899	0.9893	0.9912	0.0008	0.0028	0.002 4
512 × 512	V	0.9922	0.9917	0.9933	0.0018	−0.0014	0.0027
	D	0.9837	0.9827	0.9857	0.0041	−0.0009	−0.0005
C3	H	0.9911	0.9950	0.9897	−0.0004	0.0013	0.0009
512 × 512	V	0.9924	0.9962	0.9915	0.0009	−0.0020	0.0006
	D	0.9831	0.9907	0.9809	−0.0021	−0.0004	0.0053
C4	H	0.9868	0.9782	0.9874	0.0025	0.0008	−0.0009
512 × 512	V	0.9816	0.9704	0.9838	−0.0011	0.0004	−0.0038
	D	0.9716	0.9537	0.9739	−0.0006	−0.0003	0.0028
C5	H	0.9383	0.9466	0.9550	−0.0013	−0.0001	0.0008
512 × 512	V	0.8906	0.9057	0.9208	−0.0008	−0.0012	0.0045
	D	0.8527	0.8733	0.8942	0.0017	0.0013	0.0041
C6	H	0.9252	0.9065	0.9104	0.0026	−0.0017	−0.0005
512 × 512	V	0.9443	0.9305	0.9318	−0.0031	0.0020	−0.0004
	D	0.9160	0.8952	0.8988	0.0021	−0.0042	0.0007

**Table 3:** IE test.

Image Name	Image Size	Original P			Cipher P		
		R	G	B	R	G	B
C1	512 × 512	7.4338	7.5060	7.3521	7.9992	7.9992	7.9992
C2	512 × 512	7.1892	7.2385	6.9944	7.9994	7.9992	7.9993
C3	512 × 512	7.3493	7.6507	7.1639	7.9993	7.9994	7.9992
C4	512 × 512	7.6137	7.3056	7.6201	7.9993	7.9993	7.9993
C5	512 × 512	7.1870	7.1900	6.8601	7.9993	7.9994	7.9993
C6	512 × 512	7.8220	7.6280	7.4874	7.9994	7.9993	7.9992

**Table 4:** IE comparison.

Algorithm	Lena	Elaine	Hill
OP	7.446090	7.504811	7.476172
Proposed	7.999366	7.999410	7.999286
Ref. [5]	7.999322	7.999128	7.999317
Ref. [18]	7.999416	7.999328	7.999175
Ref. [19]	7.999336	7.999145	7.999168

**Table 5:** NPCR and UACI.

	Image Name (R, G, B)	C1	C2	C3	C4	C5	C6
		512	512	512	512	512	512
	R	99.6159	99.6208	99.6162	99.6040	99.5960	99.6006
NPCR	G	99.5899	99.5918	99.6212	99.5876	99.5953	99.6468
	B	99.5888	99.6094	99.6082	99.6002	99.6101	99.6117
	R	33.5659	33.5302	33.5485	33.5028	33.4457	33.4537
UACI	G	33.4873	33.4128	33.4624	33.4429	33.4572	33.3787
	B	33.4384	33.4583	33.5681	33.5141	33.4721	33.4792

**Table 6:** NPCR and UACI test results on different plaintext images.

Plaintext Type	Image Size	NPCR (%)	UACI (%)
All-black image	512×512	99.5922	33.4356
All-white image	512×512	99.5983	33.4076
50% Black 50% White	512×512	99.6136	33.4523

**Table 7:** U and N comparison.

Algorithm	Lena 512 × 512		Elaine 512 × 512		Hill 512 × 512	
	NPCR	UACI	NPCR	UACI	NPCR	UACI
Proposed	99.586	33.455	99.601	33.451	99.589	33.452
Ref. [5]	99.565	33.450	99.574	33.439	99.557	33.455
Ref. [18]	99.566	33.452	99.575	33.445	99.571	33.452
Ref. [19]	99.554	33.338	99.563	33.477	99.513	33.384
Ref. [29]	-	-	99.631	33.620	-	-
Ref. [30]	-	-	-	-	99.6012	33.452

## Data Availability

No new data were created or analyzed in this study. Data sharing is not applicable to this article.
